# Atopic dermatitis and association of risk for primary immune thrombocytopenia and autoimmune diseases among children: A nationwide population-based cohort study

**DOI:** 10.1097/MD.0000000000004226

**Published:** 2016-07-22

**Authors:** Chang-Ching Wei, Cheng-Li Lin, Te-Chun Shen, Jeng-Dau Tsai

**Affiliations:** a China Medical University Children's Hospital; b College of Medicine, China Medical University; c Management Office for Health Data, China Medical University Hospital; d Division of Pulmonary and Critical Care Medicine, Department of Internal Medicine; e Department of Pediatrics, Chung Shan Medical University Hospital; f Institute of Medicine, Chung Shan Medical University, Taichung, Taiwan.

**Keywords:** atopic dermatitis, autoimmune disease, population study, primary immune thrombocytopenia, retrospective cohort study

## Abstract

Primary immune thrombocytopenia (ITP) is currently defined as an acquired autoimmune disorder with persistent thrombocytopenia. However, the temporal interaction between T helper type 2 cell (Th2)-mediated allergic diseases and T helper type 1 cell (Th1)-mediated ITP remains unknown. Atopic dermatitis (AD) is considered one of the first steps in the atopic march. Herein, we conducted a population-based cohort analysis to investigate the risk of ITP in children with AD in comparison with non-AD controls. We subsequently compared the occurrence of other autoimmune diseases in ITP children in both AD and non-AD cohorts. From 2000 to 2007, 120,704 children with newly diagnosed AD and 241,408 randomly selected non-AD controls were included in the study. By the end of 2008, incidences of ITP in both cohorts and the AD cohort to non-AD cohort hazard ratios (HRs) and confidence intervals (CIs) were measured. Comparison of the occurrence of other autoimmune diseases in ITP between children with and without AD was analyzed. The incidence of ITP during the study period was 1.72-fold greater (95% CI: 1.13–2.62) in the AD cohort than in the non-AD cohort (6.96 vs 4.00 per 100,000 person-years). The risk was greatest among male children, children >2 years, those in densely populated areas, and those with white-collar parents. The HR of ITP in AD children increased significantly with the number of AD-related clinical visits (*P* < 0.001). The risk of developing ITP in the AD cohort was highest within the first 3 years after the diagnosis of AD (HR: 1.78; CI: 1.14–2.78). The AD cohort with ITP had a higher occurrence rate of other autoimmune diseases than the non-AD cohort with ITP. AD children had a greater risk of developing ITP and other autoimmune diseases. Further research is needed to clarify the role of allergy in the pathogenesis of ITP and autoimmune diseases.

## 1 Introduction

Primary immune thrombocytopenia (ITP) is the most common cause of isolated thrombocytopenia in children. ITP typically presents with petechiae, purpura, and mucous membrane bleeding, which usually develop after an upper respiratory tract infection. The peak age is 2 to 5 years.^[1,2]^ ITP is categorized as an autoimmune disorder and is characterized by persistent thrombocytopenia due to autoantibodies binding to platelets, resulting in platelet destruction in the reticuloendothelial system.^[1,2]^ Although the basic pathophysiology of ITP has been known for over 50 years, the factors responsible for triggering, or protecting against, ITP development have not been clearly established.^[1,2]^ A number of previous studies have described the activation of precursor CD4+ T lymphocytes (Th0) and T helper type 1 (Th1) cells, increased soluble interleukin (IL)-2 receptors, and increased Th1/T helper type 2 cell (Th2) ratios in patients with ITP.^[3–5]^ However, it is not clear whether these immune abnormalities play causal roles in the disease, or are simply secondary epiphenomena brought on by the inflammatory processes that are associated with ITP. In contrast to ITP, atopic dermatitis (AD) is a Th2-driven chronic relapsing inflammatory skin disease. AD is typically the first clinical manifestation of allergic disease, presenting early in infancy, followed by the development of allergic airway diseases in some children.^[6,7]^ This so-called atopic march suggests a common etiology for the different atopic diseases.^[6,7]^ Hence, AD children represent an appropriate cohort to assess the temporal interaction between Th2-mediated allergic disease and Th1-mediated ITP. Therefore, this population-based cohort study was designed to examine whether AD, as an index disease of Th2-mediated atopic diathesis, affects the development of ITP and other autoimmune diseases.

## 2 Methods

### 2.1 Data source

The National Health Insurance Research Database (NHIRD), maintained by the National Health Research Institutes, is population-based and generated from the claims data of the National Health Insurance program, a mandatory-enrollment, single-payment system created in 1995, covering over 99% of Taiwan's population (http://www.nhi.gov.tw/english/index.aspx).^[8,9]^ This study used the children file derived from the NHIRD with the information for half the population of children in Taiwan.^[10–13]^ The dataset contained the information of insurants and their medical claims, which provided a sufficient sample size for use in this study. Because of the personal electronic data-privacy regulation, the identification of each insurant was encrypted. The study was approved by the Institutional Review Board of the China Medical University Hospital (CMUH104-REC2-115). Diseases were coded based on the International Classification of Diseases, 9th Revision, Clinical Modification (ICD-9-CM).

### 2.2 Study subjects

This retrospective cohort study aimed to compare the incidence rate and relative risk (incidence rate ratio) of ITP in an AD cohort and a non-AD cohort during 2000 to 2007. Based on its chronic relapsing nature, AD was defined as at least 3 ambulatory claims in any diagnosis field or 1 inpatient claim in a primary diagnosis field with the ICD-9-CM code 691.8. A total of 120,704 patients (aged <18 years) newly diagnosed with AD between 2000 and 2007 were identified as the AD cohort. The baseline index date was the date of AD diagnosis. For each child with AD, we randomly selected 2 non-AD children, who never had ICD-9-CM code 691.8 in any diagnosis field, matched by age, sex, urbanization level, parental occupation, and baseline year. Children with missing data or those with preexisting ITP and autoimmune diseases before the baseline examination were excluded. Children with ITP can easily be detected by caregivers due to petechiae and mucosal bleeding. The majority of childhood ITP cases resolve spontaneously within 6 months and are followed at outpatient clinics; however, children with ITP are occasionally hospitalized for observation owing to the possibility of internal bleeding. Hence, ITP was defined by ICD-9-CM diagnosis code 287.3 for at least 2 ambulatory claims in any diagnosis field or 1 inpatient claim in a primary diagnosis field, excluding congenital and hereditary thrombocytopenic purpura (ICD-9-CM 287.33) and Evan syndrome (ICD-9-CM 287.32). Each child was followed up from the index date until the development of ITP, withdrawal of insurance, or conclusion of follow-up person-years on December 31, 2008.

### 2.3 Statistical analysis

The sociodemographic variables in this study were age, sex, urbanization level, and parental occupation. Urbanization level was defined according to population density, which was categorized into 4 levels: level 1 being the densest and level 4 being the least dense. All data analyses were performed using SAS software version 9.1 (SAS institute Inc., Carey, NC). Statistical significance was set at *P* < 0.05 in 2-tailed tests. The means and standard deviations (SDs) for continuous variables, and counts and percentages for categorical variables, were used to demonstrate the baseline distributions of the AD and non-AD cohorts.

Differences were examined using the Chi-square test for categorical variables, and Student *t* test for continuous variables. The Kaplan–Meier method of survival analysis was used to estimate the proportion of study subjects who did not suffer from ITP during the follow-up period for both cohorts, and the incidence densities were calculated for each cohort. The incidence rate of ITP is shown as the number of newly diagnosed ITP per person-years in both the AD and non-AD population. Person-time is the sum of individual units of time that the persons in the cohort study population had been exposed to or were at risk for the conditions of interest. Hazard ratios (HRs) and 95% confidence intervals (CIs) were calculated using multivariable Cox proportional hazard regression models, with the non-AD control cohort as the reference group, to assess the association between AD and the risk of developing ITP.

The Cox proportional hazards model was also used to estimate the HRs of ITP by the annual average AD-related medical visits. Further analysis assessed whether the association of ITP varied according to the length of the follow-up period after AD was diagnosed.

## 3 Results

**Figure 1:**
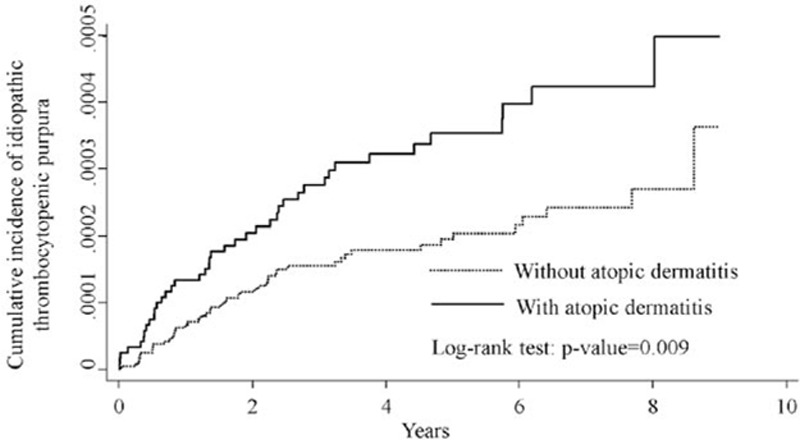
Cummulative incidence of primary immune thrombocytopenia for patients with atopic dermatitis (dashed line) or without atopic dermatitis (solid line).

This study evaluated 120,704 AD cases and 241,408 non-AD control children. With similar distributions in sociodemographic characteristics for AD and non-AD cohorts, the majority of AD cases were aged ≤2 years (48.5%), living in higher urbanization regions (35.1%), and had parental occupations of white-collar workers (66.2%; Table 1). The Kaplan–Meier analysis revealed that the ITP rate was higher in the AD cohort compared to the non-AD cohort during the observation period (*P* = 0.009, by log-rank test; Fig. 1).

**Table 1:**
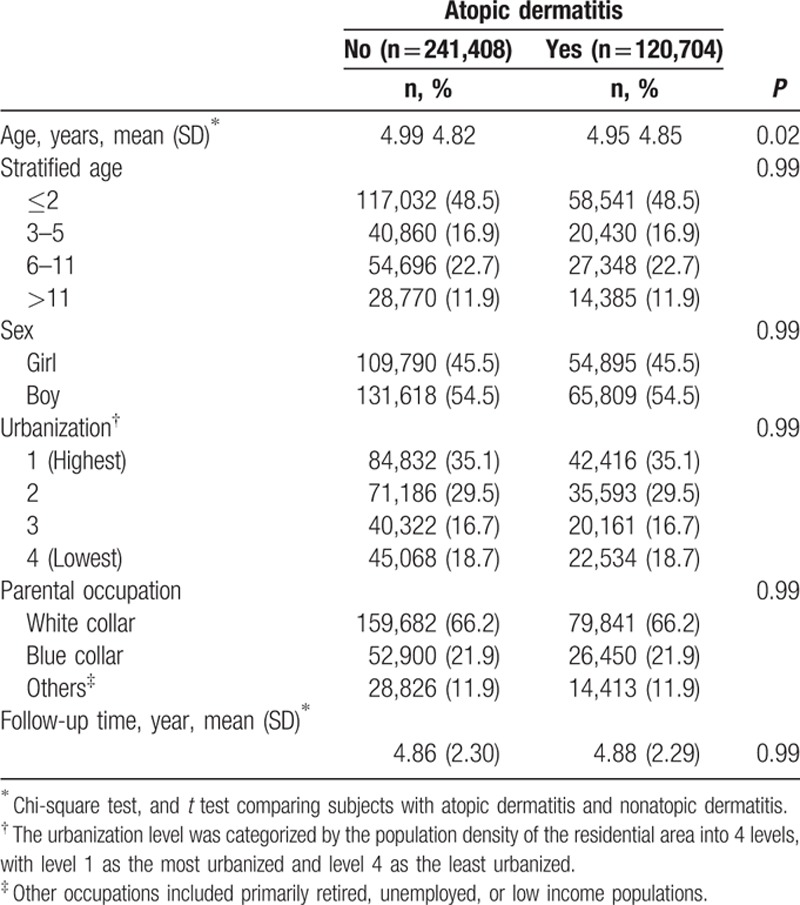
Demographics between children with and without atopic dermatitis.

The incidence densities for ITP in both cohorts, along with the AD to non-AD HRs for ITP stratified by demographic characteristics and medical utilization, were observed (Table 2). At the end of follow-up, the ITP incidence density was found to be 1.72-fold greater (95% CI: 1.13–2.62) in the AD cohort than in the non-AD cohort (6.96 vs 4.00 per 100,000 person-years). The ITP incidence density was 2.4-fold greater for AD children aged >2 years and was slightly decreased for those younger than 2 years, compared with the non-AD cohort. The risk for ITP development was approximately 8% higher for boys in both cohorts. The population density-specific HR for the AD cohort compared to the non-AD cohort was greater in densely populated areas. Compared to the non-AD cohort, the HR elevated with the frequency of AD-related medical visits per year, from 0.89 (95% CI: 0.53–1.52) for those with ≤2 visits, up to 15.5 (95% CI: 8.64–27.8) for those with ≥4 visits (Table 3).

**Table 2:**
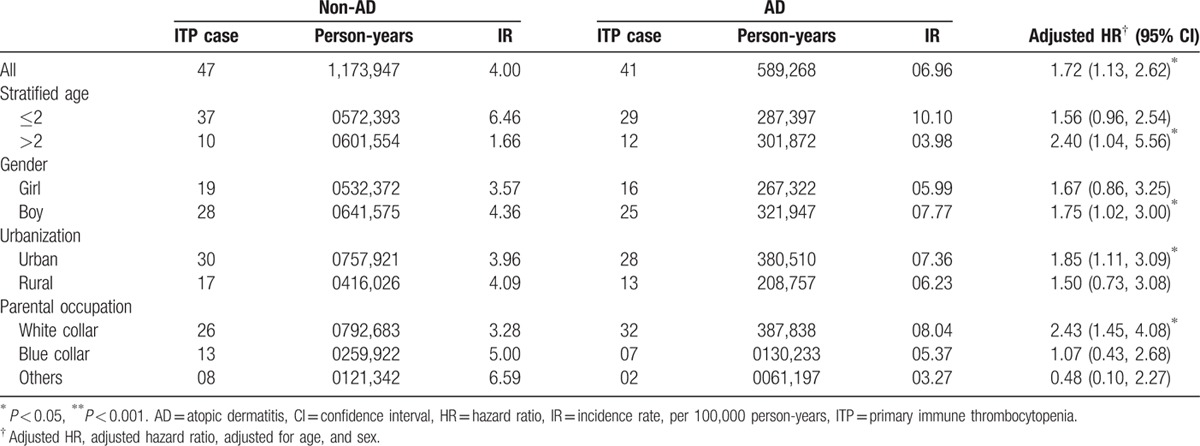
The incidence rate and risk of ITP in children with AD compared to non-AD controls stratified by demographics in Cox proportional hazard regression.

**Table 3:**
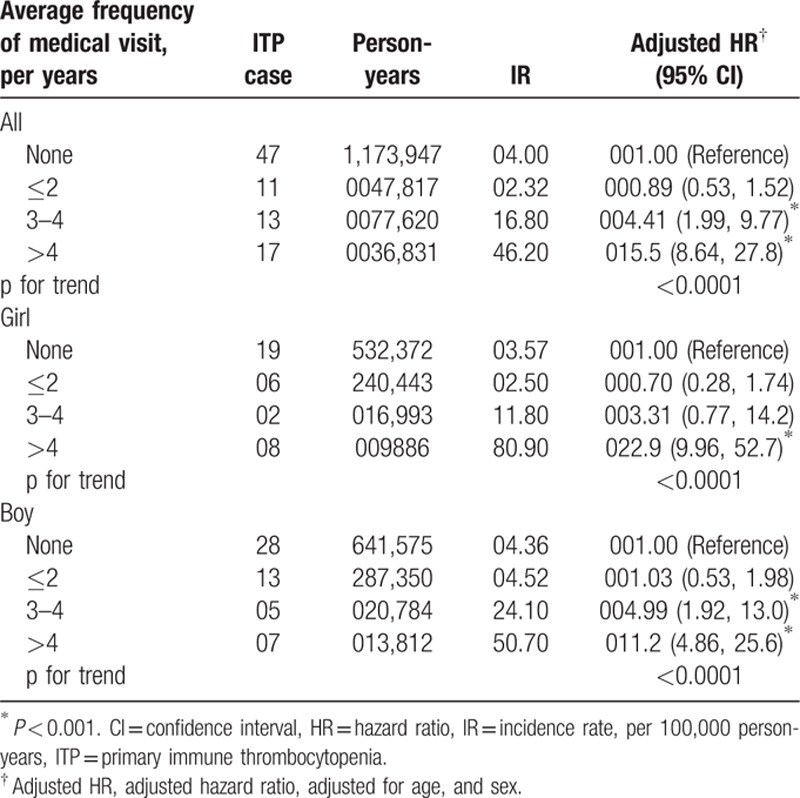
The incidence rate and risk of ITP stratified by average annual medical visits for atopic dermatitis and by both sex in Cox proportional hazard regression.

Table 4 demonstrates that AD children had a higher adjusted HR of 1.78 (95% CI: 1.14–2.78) for being diagnosed with ITP within the first 3 years after being diagnosed with AD. The adjusted HR decreased to 1.33 (95% CI: 0.37–4.70) after the 3-year follow-up period (Table 4). The AD cohort with ITP had a higher occurrence rate of other autoimmune diseases, such as autoimmune thyroid disease, vitiligo, lupus erythematosus, and type 1 diabetes, than the non-AD cohort with ITP (Table 5).

**Table 4:**
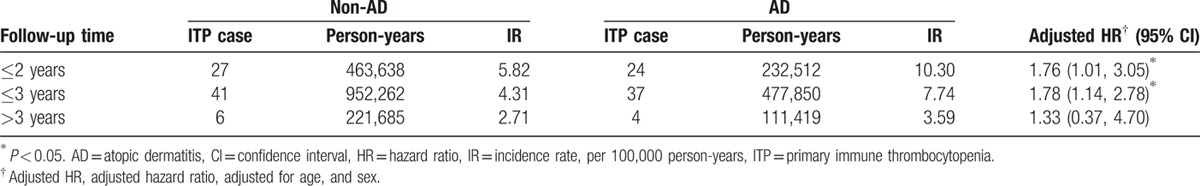
The incidence rate and risk of ITP in children with AD compared to non-AD controls, stratified by follow-up time in Cox proportional hazard regression.

**Table 5:**
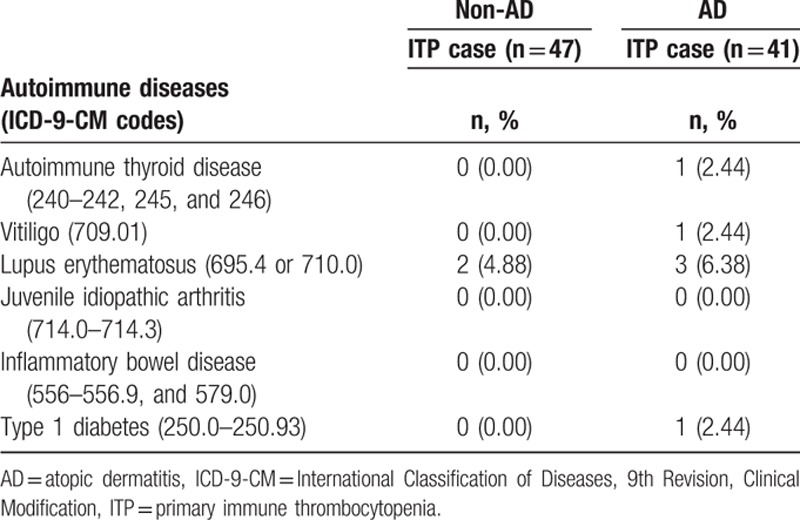
Comparison of autoimmune diseases in ITP between children with AD cohort and non-AD cohort.

## 4 Discussion

This is the first population-based cohort study to investigate the incidence of ITP in children with AD compared to a non-AD control group. Epidemiological studies have shown that ITP has an annual incidence of approximately 2.2–5.3/100,000 children.^[1,2]^ The current study revealed an annual incidence rate of ITP of 4/100,000 in non-AD children and 6.96/100,000 in AD children. In a previous registry, 78% of children with ITP were aged 0 to 7 years. In that same age category, more boys were diagnosed with ITP than girls, although the sex ratio was equal among those between the ages of 8 and 14.^[1,2]^ Our results showed boys with a higher annual incidence rate than girls in both AD (7.77/100,000) and non-AD (4.36/100,000) children. Compared to non-AD children, our results showed an increased subsequent risk of ITP in AD children. AD children who were male, older than 2 years, and living in an urban area had a higher risk of ITP. The risk also increased with an increase in AD-associated medical care, which may indicate more serious or uncontrolled skin inflammation related to AD.

AD is a chronic inflammatory skin disorder that often develops in early infancy. AD can persist beyond the childhood years and often precedes asthma and allergic rhino-conjunctivitis; as such, it represents the beginning of the atopic march.^[7]^ Therefore, the AD cohort is a useful model to study the influence of atopy on certain diseases. Children with AD were at an increased risk of developing ITP, especially those who were >2 years old. These findings may be related to the fact that the peak age of acute ITP is between 2 and 5 years of age, a period when children experience the greatest frequency of viral infections.^[14]^ We also found that greater risk of ITP for the AD cohort compared to the non-AD cohort. Microorganisms may be easily transmitted in crowding cities or densely populated areas. Hence, children living in crowding areas have greater risks to get viral infections, which may trigger the development of ITP. Recent advances in our knowledge of the pathogenesis of AD have posited a relationship between immune dysregulation and skin barrier abnormalities.^[7]^ These findings imply that AD may have a positive influence on the development of ITP, or AD and ITP may both share common early-life determinants.

ITP is a heterogeneous disease, characterized by the presence of antiplatelet antibodies and the destruction of circulating platelets.^[15]^ Historically, ITP has been considered a Th1-driven autoimmune disorder based on evidence of increased numbers of HLA-DR+ T cells, increased soluble IL-2 receptors, and Th1 cytokines in patients with ITP compared to controls. Suppressed levels of Th2 cytokines, such as IL-4 and IL-5, have been found in patients with active ITP compared to those in remission.^[4]^ In contrast to these findings, our results show an increased incidence rate and risk of ITP in Th2-mediated AD children. The mechanisms for this association may be explained by the following reasons. First, environmental factors, such as viral or bacterial infection, affect the development of allergic and autoimmune diseases.^[16]^ Second, FcγRIIb, a low-affinity IgG Fc receptor, has been shown to play a role in both allergic disease and ITP.^[17,18]^ In addition, FcγRIIIa genetic polymorphisms play a role in the pathogenesis of ITP and atopic disease.^[17,19,20]^ Third, compared to Th1 bias, Talaat et al^[21]^ found elevated Th2 cytokines (IL-4, IL-10) in patients with ITP. Finally, decreased numbers and impaired function of CD4+CD25+ regulatory T cells (Treg) have been observed in patients with ITP.^[22–24]^ The number of Treg cells is considered to be proportional to the severity of ITP, as these cells are significantly depleted in the active phase of the disease, and increased in the complete remission phase.^[22–25]^ Decreased numbers and dysfunction of Treg cells have also been reported in patients with allergic diseases.^[26–28]^ Treg cells prevent the activation and proliferation of potentially autoreactive T cells that have escaped thymic deletion in the induction and maintenance of peripheral self-tolerance.^[20]^ These previous studies imply AD and ITP may share common early-life determinants and the same immunological aberrancy.

The functional diversity of Th cells plays a crucial role in coordinating immune responses. The Th1 and Th2 paradigm originally described the reciprocal downregulation of Th1 cells by Th2 cytokines and of Th2 cells by Th1 cytokines.^[29]^ In addition, Th1 and Th2 driven immunity are involved in autoimmune and allergic diseases, respectively. Our results are in contract with the Th1/Th2 paradigm, showing that AD children had a higher subsequent risk of ITP and children with both AD and ITP had higher occurrence rates of other autoimmune diseases, such as autoimmune thyroid diseases, vitiligo, lupus erythematosus, and type 1 diabetes. Interestingly, there are several articles referring to the increased incidence of allergy among patients with autoimmune diseases, such as celiac disease, type 1 diabetes, or rheumatoid arthritis.^[30–32]^ In addition, recent studies have also reported an increased incidence of autoimmune diseases in patients with allergic diseases, such as AD and asthma.^[33–35]^ Recent advances in research of T cell biology demonstrate that T cell-dependent immunity cannot fit a simple Th1/Th2 paradigm. New Th subsets have been found distinct yet overlapping functions with Th1/Th2 cells.^[36–38]^ For example, Th17 cells can stimulate inflammatory reactions and reinforce the cellular immune response against extracellular pathogens, such as bacteria, fungi, and viruses. However, Th17 cells are also involved in the pathogenesis of autoimmune and allergic diseases.^[39–41]^ Similarly, immunosuppressive CD4+CD25+ Foxp3+Treg cells exert important effects on the maintenance of immune homeostasis and immune tolerance by producing antiinflammatory cytokines, which can inhibit both Th1 and Th2 responses.^[39–41]^ Moreover, our finding of the association of AD with multiple autoimmune diseases implies that early-life environmental and immunogenetic factors may have generalized effects on the development of both AD and autoimmune disorders.

AD is a chronic inflammatory skin disease. An impaired epidermal barrier is a key pathogenesis of atopic eczema. Biphasic cutaneous immune response was observed in AD.^[42]^ In the initiation acute phase, IL-4 production by Th2 is predominant; in the late and chronic phase, interferon-γ production by Th1 is predominant.^[42,43]^ The effect of treatment being given to AD patients has been well investigated in epidermal immunity. However, the long-term influence of the AD treatment may have an influence on Th1 and Th2 immunity is not clear. The use of emollient is recognized as a proactive treatment to maintain the integrity of epidermal barrier because an intact epidermal barrier shields most environmental stimuli from immune cells.^[44]^ Skin barrier function and epidermal immunity have been extensively studied in AD patient managed with topical treatment. Topical corticosteroids have provided effective flare control by means of their antiinflammatory, and antiproliferative actions.^[45]^ They suppress the release of inflammatory cytokines and act on a variety of immune cells, including T lymphocytes, monocytes, macrophages, dendritic cells, and their precursors.^[45]^ Topical calcineurin inhibitors inhibit of cytokine transcription in activated T cells. In only 10% of the cases, AD is so severe and nonresponsive to topical treatment that short-term systemic treatments are necessary.^[45]^ Systemic administration of corticosteroids and cyclosporine has been found to restore Th1/Th2 cytokines balance in AD patients.^[45,46]^

This study had several limitations. Some clinical presentations and a number of possible confounding variables associated with allergic diseases (e.g., severity of AD, serum IgE level, eosinophil level, and family history of allergic diseases) were not included in the database. Another limitation was the lack of data regarding genetic and environmental factors that might affect the risk of developing autoimmune and allergic diseases.

In conclusion, this population-based cohort study revealed a significantly increased incidence of ITP in children with AD and children with both AD and ITP had higher occurrence rates of other autoimmune diseases. Future studies exploring common environmental and genetic factors and aberrant immune responses related to allergic disease and autoimmune diseases are warranted.
